# An Essay on Tetanus, Founded on Cases and Experiments

**Published:** 1825-10-01

**Authors:** 


*An Essay on Tetanus, founded on Cases and Experiments.* By Joseph Swan, Member of the Royal College of Surgeons, and Surgeon to the Lincoln County Hospital. Octavo, pp. 98. London, 1825.

Tetanus is among the very many diseases of which we are nearly ignorant, both as regards the pathology and treatment. Although the facts contained in Mr. Swan's little brochure are rather too few for the foundation of a theory, yet they may become valuable by leading to further investigation in this formidable and painful disease. The first time Mr. Swan had an opportunity of making a complete dissection of a tetanic patient was in May 1823, and he then observed an unhealthy appearance of many of the ganglia of the great sympathetic nerves. He could not, however, determine, whether the said appearances were connected with the tetanic symptoms, or caused by the remedies employed—calomel, opium, and spirits of turpentine. In the following November, he dissected another body, where the person had died of tetanus—and there again he found the ganglia of the grand sympathetic exhibiting similar appearances. He, consequently, began to think, that the altered state of the ganglia might stand in the relation of cause to the disease. About this time, he found that Dr. Aronssohn, of Strasburg, had observed similar appearances—as well as the younger Andral, who discovered a great redness of the semilunar ganglia in a patient who laboured under fever, and died with symptoms of tetanus. While making some experiments on this, a third case occurred in the practice of Mr. Macauley, who permitted Mr. Swan to be present at the examination of the body, and they found appearances similar to those already alluded to above. When he had nearly finished his observations on the disease in question, he read the following opinions of Mr. Abernethy, as published in the 5th volume of the Lancet.

"When the irritable and sloughing state of the wound shall have gone off, when healthy granulations have formed, and the cicatrix is rapidly advancing, in this state tetanus may occur. How are we to account for this? I am inclined to think that it is to be accounted for in the following manner. We know that the disposition to disease occurs long before the action of the disease takes place. In the painful and bad state of the wound, the disturbed state of the nervous system has produced disease of the digestive organs, which re-acted on the nervous organs; so that the disorders have reciprocally aggravated one another; so that, at last, even though the wound is drawing to cicatrization, or has even healed, and there is no longer any local irritation, the disposition to Tetanus, from the established irritation of the cerebral and visceral functions, comes into activity. Perhaps those who know nothing of the opinions I have formed on subjects of this kind, may not thoroughly understand what I mean. I propose this as a question to surgeons, Whether the disordered state of the digestive organs, established during the irritative state of the wound, may not be the occasion of Tetanus when that irritative state has ceased?" 8.

Mr. Swan has also perceived a correspondence between his own opinions and those of Mr. Abernethey, in the work of Dr. Good—a correspondence which naturally tends to strengthen the probability of the said opinions. Conceiving it to be a matter of considerable moment to ascertain how the body is usually affected by injuries and diseases, Mr. S. has thought it necessary to premise his observations by a short enquiry into the nature of—

CONSTITUTIONAL IRRITATION.

In slight local injuries, no constitutional disturbance ensues; but, where the injury is severe, the whole body sympathises, and the functions of every part become more or less deranged —especially those of the digestive organs and circulating system. These sympathetic functional disturbances leave traces of material change after death, of which the following case is an illustration.

"Susanna Graham, a stout-looking girl, æt. seven years, was burnt by her clothes catching fire, on the 20th of August, 1823, at noon. The thighs, and arms and back, were the parts affected; but some of them appeared to be burnt deep. The skin of the abdomen was not burnt. She did not appear to suffer much pain. Mild ointment was applied to the affected parts.

"21st. At ten, A.M. she complained of pain in her belly. She had vomited several times. She wandered a little and was cold, and her face was death-like. She said she remembered me. The pulse could not be felt in either arm. Purging medicines were given her, but they did not remain in the stomach.

"In the evening the pulse was perceptible, but she appeared weaker, and was quite insensible; the pupils of the eyes were much dilated, and did not contract on the approach of light, but she could see, for she attempted to blow out the candle. Six leeches were applied to the temples. She died at one, A. M. on the 22nd.

"*Examination.*

"The lungs were very purple and loaded with blood. There were spots of ecchymosis behind the posterior mediastinum on the left side; there was a great vascularity on the outside of the aorta; there was some fluid in the pericardium.

"There was an increased redness on some parts of the omentum, but the peritoneum appeared healthy. The liver was generally pale, and especially on its concave surface. There were some spots of redness on the villous coat of the stomach, but I could not decide that they amounted to disease. About six inches of the jejunum were highly inflamed.

"All the ganglia of the grand sympathetic nerves in the chest were vascular. On the right side, the semilunar ganglion and all the rest of the ganglia in the abdomen were very vascular. On the left side, the semilunar ganglion and the first in the abdomen, formed by the continuation of the grand sympathetic nerve, were very vascular, but the others were not. The nerves of each axillary plexus were very vascular. The sciatic nerves within the pelvis and the anterior crural nerves were vascular, but not near so much so as the axillary plexus." 14.

An old woman, aged 80, fell down, on the 14th November, and injured her hip, where she complained of violent pain. Mr. Swan could not ascertain whether or not there was fracture of the neck of the thigh-bone. She had a great deal of constitutional irritation. She lingered out a month and a few days, during the latter part of which she complained of pain in the abdomen, which was very tender on pressure. She died on the 19th December. On examination, a fracture of the neck of the thigh-bone, entirely with the capsular ligament (the reflected part of which was not torn) was found, and the greater part of it united. Although it is granted that fractures of the neck of the thigh-bone may unite within the capsular ligament when the reflected part is not torn, yet when we consider the patient's age in this case, and the terrible constitutional disturbance that was going on from the date of the accident till her death, a space of only five weeks, we confess that we feel great surprise, or almost a doubt of there having been a fracture, and of its firm union by bone, under such circumstances, notwithstanding that Sir Astley Cooper acknowledged it to be such. We have seen several sections of the bones of aged people, and they very generally shew white lines running across the neck of the femur, having the appearance of former fractures united, although no such fractures had ever taken place. We should be glad to know whether Mr. Swan examined the other hip-joint, and what were the appearances there. These observations, however, are only thrown out as hints, and not, in the slightest degree, meant to question the discrimination of the able surgeons above alluded to. Mr. Swan has another preparation, taken from a woman who lived about the same time after a similar accident, and in which almost the whole of the reflected ligament was torn. Not the least union had taken place. But to return to the dissection of the old woman of 80. There was violent inflammation of the semilunar ganglia. The viscera of the chest were sound. The outside of the aorta was very vascular, as were all the intestines—bordering, in fact, on a state of inflammation. There was no other particular appearance.

Here our author relates a series of experiments made on animals, with the view of illustrating the subject under discussion. These experiments, eight or nine in number, chiefly consisted in the production of constitutional irritation in wounds of animals, by the introduction of irritating substances, as arsenic and gamboge. In almost all these experiments, the ganglia of the great sympathetic nerves were found inflamed. We now come, however, to the main part of the work, the cases of tetanus where the appearances abovementioned were found in the human subject. Previously, however, to the detail of these cases, Mr. Swan deems it necessary to answer an objection that has been made to the ganglionic appearances abovementioned. The objection and answer will be best seen by the following short extract.

"Although I am well convinced, from numerous dissections, that the ganglia of the grand sympathetic nerves have a great vascularity, induced only by several medicines and diseases, yet some are inclined to think that this appearance is present in a state of health. In answer to this opinion, I beg to observe, that I examined an executed subject immediately after it was cut down, and found the ganglia of a pearly appearance, and free from any mark of vessels carrying red blood. I have found one ganglion very red from a number of vessels filled with blood, and the corresponding one nearly white; and I have so often observed this difference, and in such a marked degree, in the same subject, as to leave no more doubt, in my mind, of its being the effect of a state of inflammation, or something bordering on that state, than there is of similar appearances constituting the inflamed conjunctiva of one eye, and the uninflamed state of the other. If a judgment is formed from the appearances presented by an injected subject, it will be erroneous, for it is always impossible to speak decidedly of the natural degree of vascularity of any part of a subject which has undergone such a preparation. The ganglia may be made red by injection, and so may the conjunctiva of the eye; we may, therefore, fairly conclude, that if the injection fills numerous vessels of the eye, which could not be observed during life, that this appearance is equally foreign to the ganglia during health." 48.

### IDIOPATHIC TETANUS.

*Case* 1. (By Mr. Macauley.) 30*th November*, 1824. Richard Ward, ætat. 10, hitherto enjoying good health, perceived a stiffness in his neck this morning, but without pain. After exposure to a bleak air this day, he was suddenly seized with a violent tetanic spasm over the greater part of the body. A quantity of rum and water was given him hot, and no medical assistance was sought till the next day, during which interval the spasm never ceased, and the symptoms were on the increase. *Dec*. 1. Mr. M. saw him at one o'clock, lying on the bed, perfectly rigid, there being complete opisthotonos. The susceptibility of the whole nervous system for conveying irritation and receiving impressions is excessive. Touching any part of the body will frequently produce a spasm. The same is the case when he attempts to swallow fluids. We need not detail the symptoms of a disease so well known. He was bled to twelve ounces, which gave great relief. A dozen of leeches were also applied to the spine, and some calomel given in sugar. Afterwards 24 leeches were ordered to the back, but before they could be applied he died. He was examined on the 2nd December, eleven hours after death.

A volvulus was found in two portions of the small intestines, the inner coats of which bore marks of great irritation. The ganglia of the great sympathetic nerve were examined minutely.

In all of them, there existed marks of decided irritation. The vessels usually pale and colourless, were injected with red blood, and the same was observed in some of the intermediate portions of nerve. "The vascularity could be distinctly traced before their removal from the body, and immersion in cold water for some time did not diminish it. The left semilunar ganglion exhibited a few vessels, but the right was injected in a beautifully minute manner, quite as much so, when seen through a magnifying glass, as the conjunctiva in a state of high inflammation." The same distinction, though not in the same degree, was observed between the two sides in all the portions of the great sympathetic nerves which were examined. In the head, the pia mater was found minutely injected—the substance of the brain healthy—some fluid in the ventricles and at the base, as well as in the theca vertebralis, which, on being opened, was observed to contain a quantity of air. The pia mater of the spinal marrow presented exactly the same appearances as that of the brain. The medulla itself was perfectly healthy.

*Case* 2. 25*th December*, 1823. F. Allen, aged 30 years, had complained of a slight pain at the stomach for several days, having previously enjoyed good health. This morning the pain extended from the stomach over the abdomen, and violent spasms came on, making the whole body completely stiff, attended by great pain. His face was now and then suffused with blood, and he had difficulty of breathing. A vein being opened, he fainted after the loss of six ounces of blood. He then drank some warm water, and took 50 drops of laudanum. He was again attacked with violent spasms of the whole body, and pain at the stomach. His jaw became occasionally locked —he complained of severe pain when pressure was made on the situation of the superior cervical ganglion of the right side —none in that of the left. Saliva kept flowing from his mouth as if he had taken mercury. The pulse was languid. After vomiting several times, the violent pain did not return, nor the tetanic spasms. Calomel and a purging mixture. By keeping up a brisk action in the bowels he soon got quite well. On this case our author observes—"from the quick termination of the violent symptoms, the following case may not be regarded as one of tetanus, but the spasms were exactly the same as I have ever witnessed in the most violent form of the complaint."

*Case* 3. This is a short notice of a case from Lobstein. A man, 47 years of age, had a fibro-cartilaginous tumour removed from the spine, to which it slightly adhered. Two years afterwards, he returned to the hospital to have another tumour removed; but, catching cold, he was seized with trismus and opisthotonos, of which he died in the course of 48 hours. On opening the body, nothing unusual was found, except a vascular net-work filled with blood on the surface of the medulla spinalis, and a quantity of serum effused within the sac formed by the dura mater. There was also distinct inflammation of the semilunar ganglia.

*Cases* 4 *and* 5. The younger Andral states that, in two individuals who died, in the year 1819, with very decided ataxo-adynamic symptoms, he found the semilunar ganglia remarkably red, apparently produced by a very minute injection of the cellular tissue, interspersed between the small grains of which the ganglia are composed. One of the individuals had presented, in the last 48 hours of his life, violent trismus, and a tetanic rigidity of the superior extremities.

*Experiment.* A small dog was destroyed by some doses of the extract of nux vomica, given at intervals during two days. The dog exhibited spasms. On examination, all the ganglia of the great sympathetic nerve were more red than usual. The pia mater of the brain and medulla spinalis were more vascular than natural. The experiment was repeated on two other dogs, but lengthened in duration. The *post mortem* results were substantially the same.

Traumatic Tetanus. Considering that this dreadful affection occasionally follows the most trifling, as well as the most serious injuries—and that, sometimes, when they are nearly healed, there is great difficulty in accounting for such occurrences.

"With a view of removing as much as possible this obscurity in the production of traumatic tetanus, I have been induced to enquire how the body is usually affected after accidents. From that enquiry I have been led to state, that when a severe injury has been received, the ganglia of the grand sympathetic nerves become irritated, and consequently the parts to which they distribute nerves. When the constitution is healthy, I believe the irritation of the ganglia goes off in a few days, and then the parts supplied by them with nerves return to a state of quietude, and again perform their healthy functions.

"When the ganglia of the grand sympathetic nerves have been thus affected, and the irritation has subsided, an unhealthy action in the wound may excite a fresh irritation in them. Or even if the wound be healed, the passions, improper food, and other causes, may continue, reproduce, or increase the disordered state of the organs receiving nerves from the ganglia, and thereby excite a fresh irritation in them.

"When the ganglia of the grand sympathetic nerves have once been in a state of irritation, I believe they are very susceptible of its renewal. When they have become again irritated, we can readily conceive that the irritation may be communicated to many of the cerebral, and all the spinal nerves, and from these to the medulla spinalis, and we can then easily comprehend how tetanic spasms may be produced." 77.

*Case.* Master Patrick, a youth 12 years of age, had the upper part of his thigh and other parts burnt, by the explosion of some squibs in his pocket, on the 5th November, 1823. He had some subsequent fever, but no vomiting nor any unfavourable symptom till the 14th November. His appetite had returned, and most of the sloughs had separated, the vacuities being nearly filled up by healthy granulations. On the last-mentioned day, he complained of pain at the stomach and sore throat, but could open his mouth very well. 15th. There came on trismus, with some slight spasms, and much difficulty of swallowing. He died the next day, with symptoms of trismus and opisthotonos.

*Examination.* The dura mater adhered to the skull more strongly than natural—the vessels of the brain were much loaded with blood—the brain itself healthy. There was some fluid in the sheath of the spinal marrow—and the vessels of the pia mater, especially near the cauda equina, were much loaded with blood. The medulla itself was not unhealthy. All the ganglia of the great sympathetic nerves in the chest were very vascular. The right semilunar ganglion was more vascular than natural; the left had a pearly appearance, and was entirely free from any mark of blood-vessels. Many of the absorbent glands in the abdomen had a remarkably red appearance.

Our author concludes his work with a few observations on some of the symptoms of tetanus, and the parts which appear to be implicated in the disease, together with an inquiry into the inferences which may be drawn from them as to the methodus medendi.

The cause of the spasms, he thinks, seldom exists in the injured part; for, although a temporary alleviation sometimes follows the removal of the part, the spasms return with the same violence as before.

The muscles affected in tetanus are supplied with nerves both from the brain and spinal marrow. Are these organs, or only their nerves, disturbed in the first instance? The functions of the brain are seldom deranged, except towards the close of the case—and not always then. On dissection, we find vascularity of the pia mater, as in many other diseases—an appearance which is probably only an *effect* of the general disturbance. The muscles of mastication and deglutition are the first affected, and yet the nerves distributed to them are principally derived from the brain. This is a very unaccountable circumstance. The increased vascularity of the pia mater of the medulla spinalis, our author looks upon only as an effect—but it shews that great irritation prevailed there.

"As the disease attacks the muscles supplied with nerves both by the brain and medulla spinalis, and neither of those organs appear in the first instance to have their functions disordered, we may fairly conclude that the cause resides in some other part.

"No other parts except the ganglia of the grand sympathetic nerves have a similar, or so free, a communication between the nerves supplying the muscles usually affected; and in all the examinations after the death, both in the human subject and the animals killed by the extract of nux vomica, I have found an increased vascularity of some of those ganglia." 89.

Mr. S. does not mean, however, to assert, that tetanus is a specific complaint, entirely seated in the ganglia of the great sympathetic nerves—but only that the ganglia are the important parts of the nervous system to which the first irritation tends, and from which it proceeds to the rest of the nervous system. We shall conclude our analysis with the following extract respecting the *methodus medendi.*

"From the appearances on dissection of patients who have died of this complaint, I cannot help concluding, that there is a state of parts bordering on inflammation, and therefore that general blood-letting is indicated. Fever, and other decidedly inflammatory symptoms, may not generally be present, yet they sometimes exist in a very great degree, as was most particularly exemplified in an interesting case* related by Mr. Earle.

"With a view of removing this congestion of the vessels of the medulla spinalis, blood should be taken from the back by leeches or cupping.

"The functions of the digestive organs are very frequently disordered, and as this state must aggravate all the other symptoms, every possible attempt should be made to restore them.

"I propose it as a question, Whether an emetic should not be given after general blood-letting has been employed, if the mouth be sufficiently open to allow of the ejection of the contents of the stomach?

"In the second case, the spasms ceased and never returned after vomiting took place. Nothing particular was ejected from the stomach,

* "Medico-Chirurgical Transactions, vol. vi. p. 93."

and therefore we cannot but suppose that the action of vomiting had a salutary effect on this organ; a circumstance very often witnessed, when every other medicine has failed to restore its disordered functions.

"After the emetic, or if this has not been used, the patient ought to be purged as soon as possible. A few doses of submuriate of mercury may be given, and any other strong purgative, until the bowels are freely emptied.

"In experiments on animals, I have found decided marks of inflammation of the ganglia of the grand sympathetic nerves produced by mercury. As there is a similar appearance of the ganglia in Tetanus, I cannot help supposing, that the use of mercury is very doubtful, if not altogether hazardous; and so many cases on record, in which it has failed to restrain the disorder, show that it cannot by any means be depended on. I am willing to believe that practitioners may have thought it beneficial, because a patient who has used it has recovered. I have seen it administered in chronic Tetanus, and the patient has got well; but the recovery was very slow; and whether it had any influence over the disease is most difficult to determine. These observations on mercury may well apply to constitutional irritation.

"When the patient has been well purged, it appears reasonable to suppose that quieting and relaxing medicines may be of use, as the pulvis ipecacuanhæ compositus given in frequent doses.

"Whether the meadow saffron would have any influence over Tetanus, I cannot determine; but from the appearances on dissection, I do not despair of a discovery of some similar medicine, which has a powerful influence in allaying irritation of the nervous system, for the removal of this dangerous and painful disease." 96.

We think Mr. Swan is entitled to the thanks of his medical brethren, for the zeal and ability which he has shewn on this, as well as upon many former occasions.

